# High Glucose Induces Oxidative Stress That Alters Glycocalyx Proteoglycan Levels in Primary Rat Retinal Microvascular Endothelial Cells and in Isolated Ophthalmic Arteries

**DOI:** 10.3390/pathophysiology31010007

**Published:** 2024-02-06

**Authors:** Ivan A. Alvarez, Minsup Lee, Randa S. Eshaq, Wendy Leskova, Norman R. Harris

**Affiliations:** 1 School of Medicine, Louisiana State University Health Shreveport, 1501 Kings Hwy, Shreveport, LA 71103, USA; iaa001@lsuhs.edu; 2 Department of Molecular and Cellular Physiology, Louisiana State University Health Shreveport, 1501 Kings Hwy, Shreveport, LA 71103, USAranda.eshaq@lsuhs.edu (R.S.E.);

**Keywords:** diabetic retinopathy, oxidative stress, glycocalyx, matrix metalloproteinase-9

## Abstract

Our purpose in this study was to identify the role played by oxidative stress in the changes to proteoglycans that occur under hyperglycemic conditions, using primary rat retinal microvascular endothelial cells (RRMEC) and cultured ophthalmic arteries. The cells and blood vessels obtained from rats were cultured in normal glucose (5.6 mM) and high glucose (25 mM) with or without N-acetylcysteine (NAC), an antioxidant. Intracellular oxidative stress was determined by measuring dihydroethidium (DHE) fluorescence and malondialdehyde (MDA)-modified protein levels. mRNA and protein levels were evaluated using quantitative real-time polymerase chain reaction and immunoblot, respectively. High glucose increased levels of glypican-1 mRNA and protein. The level of syndecan-1 mRNA also was increased, but its protein level was decreased, by high glucose. Evaluation of DHE and MDA showed that high glucose increased oxidative stress. These changes caused by high glucose were significantly reversed by NAC treatment. Matrix metalloproteinase-9 (MMP-9) levels, which increased under high glucose conditions, were suppressed by NAC treatment. Oxidative stress caused by hyperglycemia may be responsible for significant changes to the ocular endothelial glycocalyx.

## 1. Introduction

Hyperglycemia in diabetes mellitus is an independent factor that can initiate mechanisms leading to the development of diabetic retinopathy (DR) by influencing the homeostasis of retinal vascular endothelial cells [1]. DR, a major complication of diabetes mellitus, is the leading cause of blindness worldwide among working-age adults [2,3]. The disease is divided into proliferative DR and non-proliferative DR. In the proliferative stage of DR, retinal microvascular changes induced by hyperglycemia, including increased vascular permeability [4], abnormal neovascularization [5], capillary blockage [6], and cotton-wool spots [7], are hallmarks. Non-proliferative DR is an early manifestation of DR without any severe symptoms but having microvascular abnormalities. Alterations of hemodynamics and pathologic endothelial changes by hyperglycemia have been found in the early stage of experimental diabetes in animal models [8,9,10]. However, the essential molecular pathways associated with the progression of non-proliferative DR are still under investigation, including the effects of hyperglycemia-induced increases in oxidative stress and pathologic endothelial changes.

The phenomenon of oxidative stress is caused by an imbalance between the formation and removal of free radicals, which are derived from molecular oxygen. At elevated levels, reactive oxygen species (ROS) are generally considered toxic to cells and include free radicals such as superoxide anion (O2·−), hydrogen peroxide (H_2_O_2_), peroxyl radical (ROO·), and the very reactive hydroxyl radical (·OH) [11]. An imbalance in oxidative stress occurs when excessive production of ROS occurs and antioxidants (e.g., vitamins A, C, and E, glutathione, α-lipoic acid, and carotenoids), antioxidant minerals (e.g., copper and zinc), or other scavenging mechanisms fail to neutralize the free radicals [12]. Oxidative stress can damage the cells of organs targeted by diabetes, especially the retina, kidney, and heart [13,14]. The retina is vulnerable, due not only to visible and ultraviolet light, but also because the fatty acids located in the retina are easily oxidized and therefore susceptible to oxidative degradation and lipid peroxidation as the result of increased production of ROS [15].

Elevated blood glucose levels increase uptake by endothelial cells, promoting their metabolic activity, which can lead to increased production of ROS. The excessive oxidative stress caused by prolonged exposure to high glucose levels in endothelial cells is related to endothelial dysfunction [16]. The endothelial glycocalyx, located on the luminal surface of blood vessel endothelial cells, is a specialized structure with a complex meshwork of glycoproteins, proteoglycans, glycosaminoglycans, and other molecules. It plays important roles in vascular endothelial function with regards to barrier function, inhibition of blood cell adhesive interactions including thrombosis, mechanotransduction of shear forces, and providing a microenvironment for local molecular interactions. Oxidative stress can cause the degradation of the glycocalyx, leading to changes in its composition and structure. This can result in a thinner or damaged glycocalyx, which in turn affects its ability to perform its crucial functions. In the retina, damage to the endothelial glycocalyx is associated with increased vascular permeability [10], which can contribute to retinal pathologies such as DR. Loss of the endothelial glycocalyx by oxidative stress under hyperglycemic conditions may contribute to the progression of DR, but the exact relationship between hyperglycemia-induced oxidative stress and the retinal endothelial glycocalyx remains unclear. In this study, we focused on the changes to two main glycocalyx proteoglycans, glypican-1 and syndecan-1, under hyperglycemic conditions, and the effect of oxidative stress on the alterations to these proteoglycans using primary rat retinal microvascular endothelial cells (RRMEC).

## 2. Materials and Methods

### 2.1. Cell Culture

RRMEC obtained from Cell Biologics (Chicago, IL, USA) were incubated in complete media containing 5.6 mM (1 g/L) glucose. For the hyperglycemic challenge, the cells were incubated with complete media containing 25 mM (4.5 g/L) of glucose. These values of control and hyperglycemic conditions approximate the values seen in Wistar rats with/without streptozotocin administration [17] in a model of diabetes. N-Acetylcysteine (NAC, Sigma-Aldrich, St. Louis, MO, USA) was used as antioxidant.

### 2.2. Animals and Blood Vessel Tissue Culture

Male Wistar rats were obtained from Charles River (Wilmington, MA, USA) and housed under constant environmental conditions of 22 °C with a 12-h light–dark cycle. The experimental protocols described below were approved by the Institutional Animal Care and Use Committee of Louisiana State University Health Sciences Center-Shreveport and adhere to the ARVO Statement for the Use of Animals in Ophthalmic and Vision Research. The rats were euthanized with CO_2_. Ophthalmic arteries beside the optic nerve were collected and washed in PBS containing antibiotics and then incubated in the conditioned media.

### 2.3. Measurement of Oxidative Stress

Intracellular oxidative stress was examined using dihydroethidium (DHE, Sigma-Aldrich), a fluorescent dye that detects reactive species including hydrogen peroxide and superoxide. Briefly, cells incubated under the desired conditions in a 96-well black plate were further incubated with 10 µM DHE for 30 min at 37 °C. The relative level of oxidized DHE florescence was measured using an Agilent BioTek Gen5 microplate reader (Winooski, VT, USA). The cells incubated in 6-well plates under the same conditions were fixed with paraformaldehyde for 10 min and immersed with Fluorescent-G containing DAPI (Nikon, Tokyo, Japan). Intracellular oxidized DHE florescence was captured using a Nikon Eclipse E600 fluorescence microscope (Tokyo, Japan). The relative level of intracellular malondialdehyde (MDA)-modified protein was measured by a modified immunoblot method running whole cell lysates into a stacking gel (pH 6.8) at 100 V for 10 min followed by transfer of a thin line onto a finger-sized nitrocellulose membrane at 250 mA for 90 min. Anti-MDA antibody (abcam, Cambridge, MA, USA) was used for detection of the proteins. Total protein by ponceau staining from the same membrane was used for normalization of the data.

### 2.4. Immunoblot Analysis

The cells or blood vessels incubated with conditioned media for 3 days were washed with ice-cold PBS and then lysed in radioimmunoprecipitation assay buffer. After centrifugation at 14,000 rpm for 20 min at 4 °C, supernatant containing the whole cell lysate was collected. Protein concentrations in the samples were determined using the bicinchoninic acid assay (Thermo Fisher Scientific, Waltham, MA, USA). Aliquots of protein were denatured with Laemmli sample buffer (Bio-Rad, Hercules, CA, USA) containing 2.5% β-mercaptoethanol (Bio-Rad) for 10 min at 100 °C. Equal volumes of protein were separated by sodium dodecyl sulfate-polyacrylamide gel electrophoresis and transferred onto a nitrocellulose membrane. The membrane was then blocked with protein-free T20 blocking solution (Thermo Fisher Scientific) for 1 h. The membranes were incubated with primary antibodies for glypican-1, MMP-9 (Santa Cruz Biotechnology, Dallas, TX, USA) or syndecan-1 (abcam, Cambridge, UK) overnight. The blots were treated with horseradish peroxidase-conjugated secondary antibodies for rabbit or mouse IgG (Jackson ImmunoResearch Laboratories, West Grove, PA, USA) for 2 h. Immune complexes were detected using Clarity Western enhanced chemiluminescence substrate (Bio-Rad) and captured using a ChemiDoc Image acquisition system (Bio-Rad). Densitometric analyses of the results were performed using ImageJ (v1.52a, NIH, Bethesda, MD, USA). Relative levels of protein expression were normalized with β-actin (Santa Cruz Biotechnology) from the same membrane.

### 2.5. Total RNA Isolation, Reverse Transcription, and Quantitative Real-Time Polymerase Chain Reaction (qRT-PCR)

Total RNA was isolated from the incubated cells using QIAzol lysis reagent (Qiagen, Germantown, MD, USA) according to the manufacturer’s protocol. Five micrograms of total RNA were reverse transcripted to cDNA using GoScript Reverse transcriptase (Promega, Madison, WI, USA) with oligo dT primer. Analysis of cDNA to determine mRNA expression levels was carried out using a Bio-Rad CFX Fast Real-Time PCR System with iTaq Universal SYBR Green Supermix (Bio-Rad). Primers used for qRT-PCR are listed in Table 1. All data were analyzed using the 2^−ΔΔCt^ method. Peptidylprolyl isomerase A (Ppia) was used as an internal control.

### 2.6. Statistics

Analyses of statistical data were performed with GraphPad Prism 10 software (GraphPad, Boston, MA, USA) using one-way ANOVA with Newman–Keuls post hoc tests. Group data are presented as means ± standard error, with *p* < 0.05 considered statistically significant.

## 3. Results

### 3.1. Effects of High Glucose on Glycocalyx Proteins and Oxidative Stress in RRMEC

**Figure 1:**
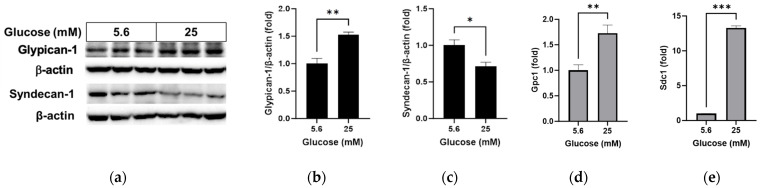
Effect of high glucose on glycocalyx in RRMEC. The relative protein expression levels of glypican-1 and syndecan-1 were normalized to β-actin expression using ImageJ (**a**–**c**). The relative mRNA expression levels of glypican-1 (**d**) and syndecan-1 (**e**) were normalized to *Ppia1* expression. *n* = 3–6; means ± SE. * *p* < 0.05, ** *p* < 0.01, and *** *p* < 0.001.

**Figure 2:**
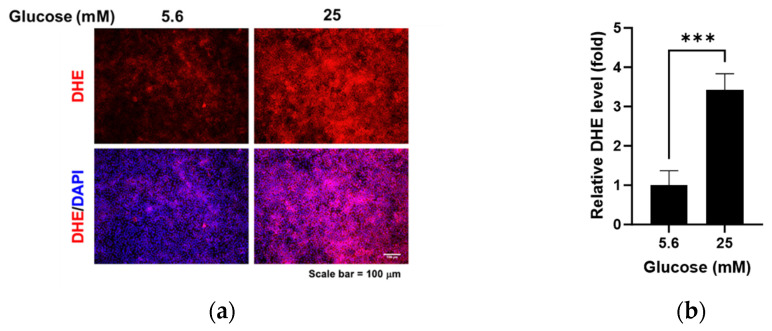
Effect of high glucose on oxidative stress in RRMEC. The representative images of DHE in RRMEC were captured using a fluorescence microscope (**a**). The relative level of DHE was measured using a microplate reader (**b**). *n* = 3–7; means ± SE. *** *p* < 0.001.

We compared the changes in glycocalyx proteins glypican-1 and syndecan-1 and oxidative stress levels under normal glucose and high glucose conditions. High glucose led to increased glypican-1 levels (Figure 1a,b; *p* < 0.01) and decreased syndecan-1 levels (Figure 1a,c; *p* < 0.05), whereas the mRNA levels of glypican-1 (Figure 1d; *p* < 0.01) and syndecan-1 (Figure 1e; *p* < 0.001) were higher under high glucose conditions compared to normal glucose conditions. The florescence signal of oxidized DHE, a marker for oxidative stress, was captured using fluorescence microscopy (Figure 2a) and relative levels were measured using a microplate reader (Figure 2b). DHE levels significantly increased in the RRMEC incubated with high glucose (*p* < 0.001).

### 3.2. Effect of NAC on the High Glucose-Increased Oxidative Stress in RRMEC

**Figure 3:**
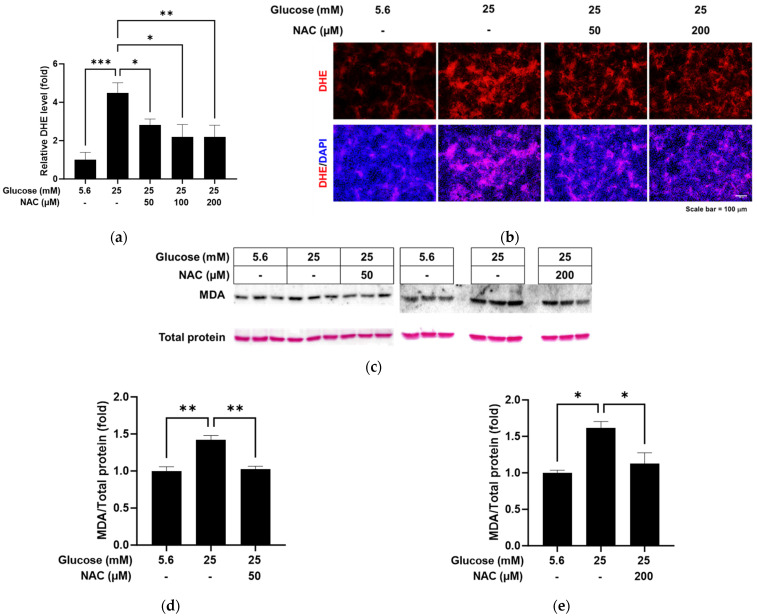
Effect of NAC treatment on the high glucose-induced oxidative stress in RRMEC. The relative level of DHE was measured using a microplate reader (**a**). The representative images of DHE in RRMEC were captured using a fluorescence microscope (**b**). The relative MDA-modified protein levels were normalized to total protein level using ImageJ (**c**–**e**). *n* = 3–8; means ± SE. * *p* < 0.05, ** *p* < 0.01, and *** *p* < 0.001.

We evaluated the ability of the antioxidant NAC to reduce the oxidative stress caused by high glucose in RRMEC. The relative florescence level of oxidized DHE was found to be significantly decreased by NAC treatment in RRMEC incubated with high glucose (Figure 3a; *p* < 0.05 at 50 and 100 µM and *p* < 0.01 at 200 µM), with corresponding images captured using fluorescence microscopy (Figure 3b). MDA is a lipid peroxidation product often used as an oxidative stress marker. Relative MDA-modified protein levels, which were increased by high glucose in RRMEC, were significantly decreased by NAC treatment at a low concentration (50 µM, *p* < 0.01) and a high concentration (200 µM, *p* < 0.05) (Figure 3c–e).

### 3.3. Effect of NAC on the Alterations Caused by High Glucose in RRMEC

**Figure 4:**
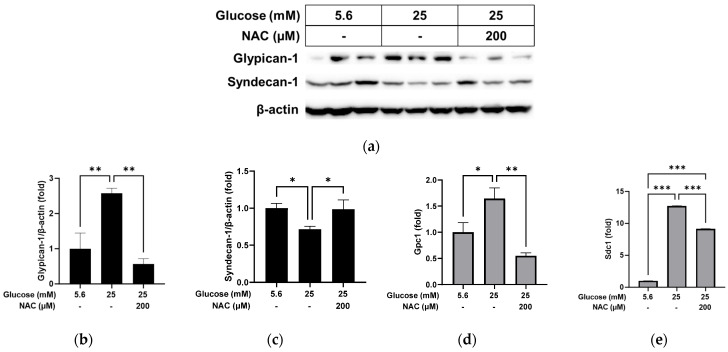
Effect of NAC treatment on the high glucose-induced changes to glypican-1 and syndecan-1 in RRMEC. The relative protein expression levels of glypican-1 and syndecan-1 were normalized to β-actin expression using ImageJ (**a**–**c**). The relative mRNA expression levels of glypican-1 (**d**) and syndecan-1 (**e**) were normalized to *Ppia1* expression. *n* = 3–6; means ± SE. * *p* < 0.05, ** *p* < 0.01, and *** *p* < 0.001.

We further examined whether treatment with the antioxidant NAC could reverse the changes caused by high glucose in RRMEC. As shown in Figure 4, the protein level of glypican-1 in RRMEC increased by high glucose was significantly decreased by NAC treatment (Figure 4a,b; *p* < 0.01), while the opposite was observed for the syndecan-1 protein level (Figure 4a,c; *p* < 0.05). mRNA levels of glypican-1 (Figure 4d) and syndecan-1 (Figure 4e) increased by high glucose were significantly reduced by NAC treatment (*p* < 0.01 for glypican-1; *p* < 0.001 for syndecan-1). The cells treated with NAC under high glucose conditions had a higher expression level of syndecan-1 mRNA compared to the cells incubated with normal glucose (Figure 4e; *p* < 0.001).

### 3.4. Effect of NAC on Protein Levels in RRMEC Incubated with Normal Glucose

**Figure 5:**
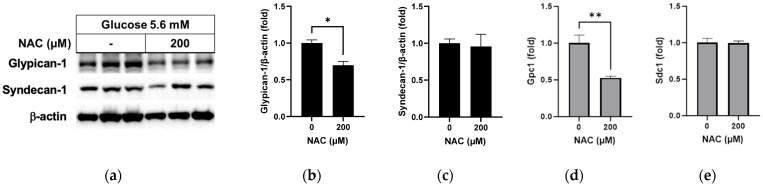
Effect of NAC treatment in normal glucose media on glypican-1 and syndecan-1 in RRMEC. The relative protein expression levels of glypican-1 and syndecan-1 were normalized to β-actin expression using ImageJ (**a**–**c**). The relative mRNA expression levels of glypican-1 (**d**) and syndecan-1 (**e**) were normalized to *Ppia1* expression. *n* = 3–6; means ± SE. * *p* < 0.05 and ** *p* < 0.01.

In a test of the effect of NAC under normal glucose conditions, the glypican-1 protein level was found to have decreased significantly following NAC treatment compared to non-treatment (Figure 5a,b; *p* < 0.05), with a significant decrease in the mRNA level (Figure 5d; *p* < 0.01). No differences were observed in syndecan-1 protein (Figure 5a,c) or mRNA (Figure 5e) levels following NAC treatment under normal glucose conditions.

### 3.5. Effect of NAC on MMP-9 Levels in RRMEC Incubated with Normal and High Glucose

**Figure 6:**
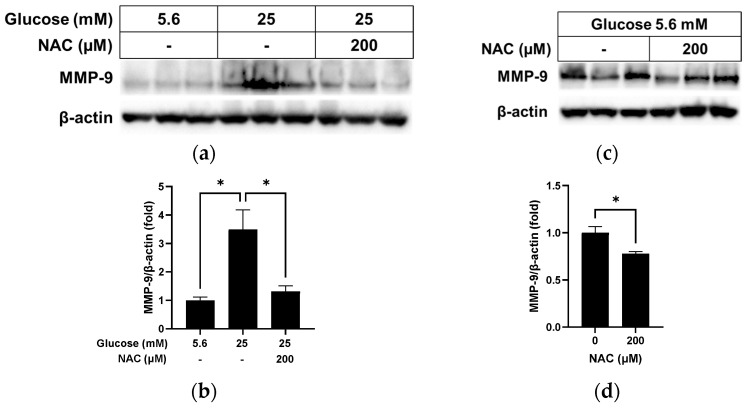
Effect of NAC treatment on MMP-9 in RRMEC maintained in high glucose and normal glucose media. The relative protein expression levels of MMP-9 were normalized to β-actin expression using ImageJ in high glucose media (**a**,**b**) and normal glucose media (**c**,**d**). *n* = 3; means ± SE. * *p* < 0.05.

MMP-9 is associated with the loss of cellular syndecan-1 [18]. As shown in Figure 6a,b, the MMP-9 protein level, which was significantly higher in RRMEC incubated with high glucose compared to those incubated with normal glucose (*p* < 0.05), decreased significantly following NAC treatment (*p* < 0.05). Under normal glucose conditions (Figure 6c,d), NAC treatment slightly reduced the MMP-9 protein level in RRMEC (*p* < 0.05).

### 3.6. Effect of High Glucose and NAC on Ophthalmic Arteries

**Figure 7:**
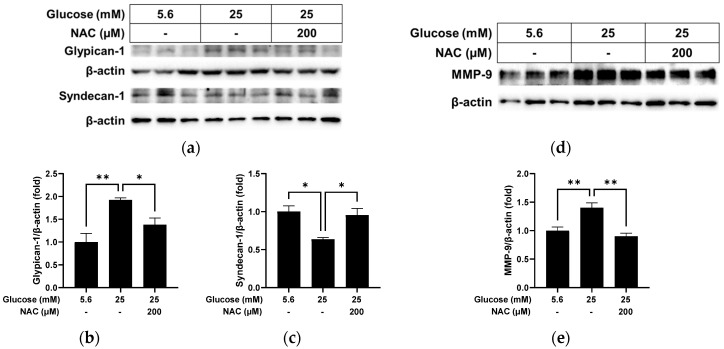
Effect of NAC treatment on the high glucose-induced changes in cultured ophthalmic arteries. The relative protein expression levels of glypican-1, syndecan-1 (**a**–**c**), and MMP-9 (**d**,**e**) were normalized to β-actin expression using ImageJ. *n* = 3; means ± SE. * *p* < 0.05 and ** *p* < 0.01.

We further evaluated the effect of NAC on the changes caused by high glucose in cultured blood vessels using ophthalmic arteries collected from rats. We found increased levels of glypican-1 (*p* < 0.01) with reduced levels of syndecan-1 (*p* < 0.05) after incubation with high glucose in cultured vessels (Figure 7a–c). NAC treatment in cells under high glucose conditions led to reduced levels of glypican-1 (*p* < 0.05) and increased levels of syndecan-1 (*p* < 0.05) in cultured blood vessels. MMP-9 levels (Figure 7d,e), which were increased by high glucose compared with normal glucose (*p* < 0.01), were also reversed by NAC treatment (*p* < 0.01) in cultured blood vessels.

## 4. Discussion

The pathology of non-proliferative DR is imperative to understand in the pursuit of strategies to prevent progression to the advanced stages of DR associated with permanent vision loss. In our effort to identify the pathogenesis of non-proliferative DR, we have demonstrated alterations of retinal hemodynamics including decreased retinal blood flow and glycocalyx thickness under hyperglycemic conditions in the early stage of experimental diabetes in Wistar rats and Ins2^Akita^ mice [8,9,10,19]. Additionally, loss of the retinal glycocalyx causes increased vascular leakage in the mouse retina [10], with increased permeability a major pathology in the progression of DR. In diabetic Wistar rats, we have found no change in retinal 4-HNE, which is an oxidative stress marker; however, NADPH oxidase, an oxidative stress producer, was significantly higher in the diabetic retinas [19] with excessive oxidative stress having been thoroughly examined and firmly associated with cardiovascular and atherosclerotic manifestations of DM [20,21,22,23]. NAC, a non-vitaminic antioxidant, has been shown to have significant effects on diabetes-induced peripheral neuropathy [24] and to decrease the thrombotic risk in patients with chronic diabetes [25]. In addition, NAC protects the glycocalyx against oxidative stress caused by heat shock or H_2_O_2_ administration in human umbilical vein endothelial cells [26]. These previous studies suggest that hyperglycemia-induced oxidative stress may cause damage to the retinal endothelial glycocalyx. However, the role played by hyperglycemia-induced oxidative stress in the pathogenesis of retinal endothelial cell glycocalyx has yet to be determined.

Hyperglycemia in DM is caused by insulin deficiency or increased insulin resistance, which regulates glucose uptake and use through the glucose transporter (GLUT). However, in vascular endothelial cells, GLUT 1 and 3, which are insulin-independent GLUT, are responsible for up to 60% of endothelial glucose uptake [27]. As a result, endothelial cell glucose uptake is rapid in hyperglycemia. Intracellular glucose enters the glycolytic pathway, which can increase the ROS production that contributes to endothelial dysfunction and microvascular pathology in DM. In this study in RRMEC, we found that high levels of glucose increased intracellular levels of ROS and MDA and that treatment with NAC under high glucose conditions reduced the production of ROS and MDA.

The elevated intracellular ROS level is caused by an imbalance between the production of ROS and the efficacy of scavengers such as catalase, glutathione, and superoxide dismutase (SOD). Administration of SOD and catalase in vivo have been shown to prevent degradation of the endothelial surface layer and endothelial–platelet adhesion induced by oxidized lipoprotein, which is a product of lipid peroxidation, suggesting that antioxidant enzymes could have an important role in the protection of the endothelial glycocalyx and from oxidative stress-induced damage [28]. Although endothelial cells promote the expression of antioxidant enzymes as a response to the increased oxidative stress caused by high levels of glucose, the response is not always adequate to achieve a balance between ROS and the antioxidants [29]. Interestingly, among the SOD family, the soluble form of SOD (extracellular SOD or SOD3) has a binding site for heparan sulfate [30], and protects the extracellular matrix against oxidative stress by binding the extracellular heparan sulfate domain [31]. Genetic mutations in *SOD3* cause loss of affinity for heparan sulfate and the endothelial cell surface [32,33] and are associated with polyneuropathy, cardiovascular disease, myocardial infarction, and DM [34]. Heparan sulfate is abundant in the glypicans; therefore, increasing heparan sulfate proteoglycans, including glypican-1, might contribute to the reduction of ROS. In our study, while the protein level of glypican-1 was increased by high glucose, treatment with NAC in cultured blood vessels and RRMEC under high glucose conditions suppressed glypican-1 protein levels. Glypican-1 was highly expressed at the mRNA level in RRMEC under high glucose conditions. The mRNA level of glypican-1 was decreased by NAC treatment under both high glucose and normal glucose conditions, suggesting that glypican-1 could be upregulated to protect endothelial cells against increased oxidative stress.

The mRNA level of syndecan-1, with the protein having extracellular attachment sites for heparan sulfate and chondroitin sulfate, was also increased by high glucose in RRMEC, but it was decreased at the protein level in cultured blood vessels and RRMEC. NAC treatment recovered the loss of syndecan-1 protein caused by high glucose in cultured blood vessels and RRMEC, but only partially suppressed the high glucose-induced overexpression of syndecan-1 mRNA in RRMEC. Syndecan-1 plays important roles in endothelial function. As a transmembrane protein, syndecan-1 modulates binding of other receptors to their ligands on the endothelial cell surface. It also controls integrin activation. Synstatin, a peptide inhibitor of syndecan-1, suppresses endothelial cell migration and angiogenesis under vascular endothelial growth factor (VEGF) stimulation through incorporative communication between integrin, insulin-like growth factor 1 receptor, and VEGF receptor 2 [35], indicating that loss of syndecan-1 caused by hyperglycemia-induced oxidative stress may be involved in retinal endothelial dysfunction and microvascular abnormalities.

The glycocalyx can be degraded through post-translational modification by several proteinases including matrix metalloproteinases (MMPs) and A disintegrin and metalloproteinases (ADAMs). Chemical inhibition or genetic knockout of tumor necrosis factor (TNF)-α receptor 1, ADAMs, and MMPs prevents the loss of endothelial glycocalyx components in in vivo or in vitro stimulations by lipopolysaccharide (a strong inflammatory mediator) or TNF-α (a pro-inflammatory cytokine) [36,37,38]. MMP-9, a collagenase, plays a key role in extracellular remodeling, and is often involved in vascular pathologic processes such as atherosclerosis, endothelial mesenchymal transition, angiogenesis, permeability, and inflammation. MMP-9 is upregulated in DM, and high glucose has been shown to induce MMP-9 in endothelial cells [23,39]. In addition, MMP-9 has been shown to be associated with the loss of syndecan-1 [18]. In RRMEC and cultured blood vessels in our study, high glucose induced MMP-9, while subsequent treatment with NAC treatment reduced the MMP, suggesting that MMP-9 potentiates the degradation of syndecan-1 protein in retinal vascular endothelial cells.

In summary, high levels of glucose induced oxidative stress in RRMEC. The endothelial proteoglycans glypican-1 and syndecan-1 were upregulated at the translational level by high glucose in RRMEC. At the protein level, glypican-1 was highly expressed but syndecan-1 was suppressed by high glucose in cultured blood vessels and RRMEC. The level of MMP-9 was increased by high glucose in cultured blood vessels and RRMEC. Antioxidant NAC treatment reversed the high glucose-induced changes in cultured blood vessels and RRMEC. Oxidative stress caused by hyperglycemia may have a significant effect on the alteration of the endothelial glycocalyx in the retina. This finding supports the need for further research on the use of antioxidants such as NAC to treat or slow the progression of diabetic retinopathy.

## Figures and Tables

**Table 1:** Primer information.

Gene Symbol	Accession No.	Gene Name	Primer Pair (5′ to 3′)
Gpc1	NM_030828.2	Glypican 1	GCTGCTGGAATGGGATTT
			ATGTCCACCTCCACTTCA
Ppia	NM_017101.1	Peptidylprolyl isomerase A	TGTGGCCCTCCTACATAAA
			AGTAGGAGACTAACCACGTG
Sdc1	NM_013026.2	Syndecan 1	CCAAATCCGGACACCAAAGGGCACCAAACAGATAGTC

## Data Availability

Data will be made available upon request.
